# Finger on the Pulse: Pumping Iron into Chickpea

**DOI:** 10.3389/fpls.2017.01755

**Published:** 2017-10-13

**Authors:** Grace Z. H. Tan, Sudipta S. Das Bhowmik, Thi M. L. Hoang, Mohammad R. Karbaschi, Alexander A. T. Johnson, Brett Williams, Sagadevan G. Mundree

**Affiliations:** ^1^Centre for Tropical Crops and Biocommodities, Queensland University of Technology, Brisbane, QLD, Australia; ^2^School of Biosciences, University of Melbourne, Melbourne, VIC, Australia

**Keywords:** pulse biofortification, iron, genetic modification, crop improvement, chickpea

## Abstract

Iron deficiency is a major problem in both developing and developed countries, and much of this can be attributed to insufficient dietary intake. Over the past decades several measures, such as supplementation and food fortification, have helped to alleviate this problem. However, their associated costs limit their accessibility and effectiveness, particularly amongst the financially constrained. A more affordable and sustainable option that can be implemented alongside existing measures is biofortification. To date, much work has been invested into staples like cereals and root crops—this has culminated in the successful generation of high iron-accumulating lines in rice and pearl millet. More recently, pulses have gained attention as targets for biofortification. Being secondary staples rich in protein, they are a nutritional complement to the traditional starchy staples. Despite the relative youth of this interest, considerable advances have already been made concerning the biofortification of pulses. Several studies have been conducted in bean, chickpea, lentil, and pea to assess existing germplasm for high iron-accumulating traits. However, little is known about the molecular workings behind these traits, particularly in a leguminous context, and biofortification via genetic modification (GM) remains to be attempted. This review examines the current state of the iron biofortification in pulses, particularly chickpea. The challenges concerning biofortification in pulses are also discussed. Specifically, the potential application of transgenic technology is explored, with focus on the genes that have been successfully used in biofortification efforts in rice.

## Introduction

The current world population stands at an estimated 7.3 billion (United Nations, 2015) and is projected to increase by 2 billion over the next four decades. Concomitant to this growth is the challenge of providing sustenance amidst dwindling resources. Currently food production is adequate at approximately four billion metric tons per annum, yet in spite of this, about 870 million people still suffer from chronic malnutrition due to factors like unequal distribution, wastage and poor diets (FAO, 2012; IMECHE, 2013).

Malnutrition, as defined by the World Health Organization (WHO), is “the cellular disparity amid the supply of energy, nutrients and the body's demand for them to ascertain maintenance, growth and specific functions” (Batool et al., 2013). It refers to both the insufficient and excessive intake of nutrients (both macro and micro) and as such covers not only food shortage but also obesity. Undernourishment can be classified categories: protein-energy malnutrition and micronutrient deficiency. As the names suggest, the former refers to inadequate calorie or protein intake while the latter to the lack of essential micronutrients such as vitamin A, iodine, zinc, and iron (Batool et al., 2013).

While both pose significant risks to health and negatively affect overall productivity and quality of life, micronutrient deficiency, also known “hidden hunger,” is perhaps the more pervasive and lethal due to the lack of visible effects. It is consequently more difficult to identify and tackle, and afflicts both developing and developed nations.

Among the various kinds of micronutrient deficiencies, iron deficiency is the most prevalent, afflicting more than two billion individuals worldwide (WHO, 2008). It has been identified as the greatest contributor to anemia, accounting for 66.2% of cases globally (Alvarez-Uria et al., 2014). The extent of its impact is such that the terms are used interchangeably and the prevalence of anemia is used as a measure for the more specific iron deficiency anemia (IDA) (WHO, 2001). IDA can be attributed to three main factors—increased iron requirement (e.g., growth and pregnancy), poor absorption, and inadequate dietary intake. The recommended values for daily iron intake varies depending on the gender and developmental stage (Table 1), and insufficient intake impedes the formation of biologically important compounds, most notably heme, resulting in anemia. Symptoms include fatigue, loss of energy, and dizziness, all of which diminish the work capacity of the individual. Iron deficiency also results in poor pregnancy outcomes and impediment of physical and cognitive development, thereby increasing the risk of morbidity in children (WHO, 2008).

**Table 1:** Recommended Dietary Allowances (RDAs) for iron (Trumbo et al., 2001).

**Age**	**Male (mg)**	**Female (mg)**	**Pregnancy (mg)**	**Lactation (mg)**
Birth to 6 months	0.27			
7–12 months	11			
1–3 years	7			
4–8 years	10			
9–13 years	8			
14–18 years	11	15	27	10
19–50 years	8	18	27	9
51+ years	8			

This presents a problem of great economic and social significance, particularly in developing countries where approximately 50% of pregnant women and 40% of preschool children suffer from IDA (WHO, 2008). The consequences manifest not only in the form of lives lost, but also in a rising generation of individuals afflicted with developmental complications. The significance of this issue has been understood by various governments, and through both nutrition and non-nutrition based interventions, considerable progress has been made in reducing IDA, particularly in South Asia, East Asia, Southeast Asia, and Eastern sub-Saharan Africa (Kassebaum, 2016). However, while a global decrease in the prevalence of severe anemia cases was observed between 1990 and 2013, the number of mild to moderate cases has increased (Table 2), and a prevalence rate below 10% has yet to be seen in any country (Kassebaum, 2016).

**Table 2:** Prevalence of anemia between 1990 and 2013 (Kassebaum, 2016).

		**Prevalent cases of anemia**		
	**Severity of anemia**	**1990**	**2013**	**Difference between 1990 and 2013 (%)**
Global	Mild	839,101,225	950,135,191	13.23
	Moderate	901,120,023	905,501,751	0.49
	Severe	88,720,928	75,565,628	−14.83
Developing countries	Mild	717,671,655	816,531,244	13.78
	Moderate	803,792,125	809,200,194	0.67
	Severe	82,149,100	69,480,441	−15.42
Developed countries	Mild	121,429,570	133,603,946	10.03
	Moderate	97,327,897.80	96,301,556.30	−1.05
	Severe	6,571,827.50	6,085,186.70	−7.40

Out of the three major risk factors contributing to IDA, the issue of dietary intake is the most feasible to address on a large scale. Efforts to remedy the problem include changes in policy, education, and food-based strategies. The latter can, in turn, come in various forms such as dietary diversification, food fortification, and supplementation, the definitions and examples of which are illustrated in Table 3. More specific details and an overview of the strategies can be found in the published guidelines by the WHO and FAO (2006).

**Table 3:** The main food-based strategies to combat iron deficiency (WHO and FAO, 2006).

	**Dietary diversification**	**Food fortification**	**Supplementation**
Definition	Inclusion and increased intake of foods rich in the target nutrient	Improvement of food nutritional quality via enhancing target nutrient content	Intake of moderately large doses of target nutrient. Can be done via oral (e.g., pills, capsules, or syrups) or intravenous routes
Examples	Leafy vegetables Pulses Red meat Seafood	Iron-fortified cereals, flour, and bread	Ferrous salts (e.g., ferrous sulfate, ferrous gluconate, ferrous fumarate) Iron dextran Iron sucrose

To summarize, each strategy has its own unique advantages and several studies have proven their effectiveness in alleviating IDA (Baltussen et al., 2004; Gera et al., 2012; Rao et al., 2013). While that efficacy may vary across different temporal and spatial scales, the strategies can be used in concert to greater effect. For instance, dietary diversification can still be used where supplementation or food fortification strategies may not due to lack of suitable infrastructure or distribution networks. However, it in turn, is subject to local environmental conditions and resource availability.

Concerning the aforementioned strategies and their application, the issue of accessibility has been noted to be a major limitation (WHO and FAO, 2006). With the food fortification and supplementation schemes in particular, the recurring costs associated with processing and distribution can be prohibitive and beneficiaries are limited to those who can afford it. Such measures are therefore unfeasible for the low-income demographics that, incidentally, have the greatest need. The challenge then is to develop a sustainable, cost-effective means to deliver the required nutrients to the vulnerable parties.

One such means is biofortification, which can generally be defined as the enhancement of nutritional quality in the edible portions of food crops during plant growth (HarvestPlus, 2015b; WHO, 2016). Given that the process of plant nutrient accumulation is a complex interplay between genetics, environmental and management factors, the precise definition of the term “biofortification” may vary depending on the scope of the means (HarvestPlus, 2015b; WHO, 2016). For the purpose of this review which focuses on the genetic aspect, the term “biofortification” shall be used to refer solely to the generation of self-fortifying plants, to the exclusion of agronomic interventions. Such agronomic interventions include fertilizer application or bacterial inoculation, which can be used in conjunction with biofortified crops. Fertilizer application has been demonstrated to increase iron accumulation, though the degree of which varies between studies (Pahlavan-Rad and Pessarakli, 2009; Cakmak et al., 2010; Zhang et al., 2010; Aciksoz et al., 2011). The use of bacterial inoculation on the other hand, has been met with some success, though the choice of strains used may depend on the environmental conditions (Mishra et al., 2011; Rana et al., 2012; Sharma et al., 2013).

## Biofortification as a means of alleviating global iron deficiency

Biofortification emerged within the last two decades as an approach to combat micronutrient deficiency. While it cannot be considered a cure-all to micronutrient deficiency, it alleviates the problem by complementing existing strategies like the aforementioned ones of dietary diversification, fortification, and supplementation. With the one-time cost of development thoroughly compensated by the long term benefits, biofortification presents a sustainable means of delivering the needed micronutrients across large spatial and temporal scales (Nestel et al., 2006; Horton et al., 2008; De Moura et al., 2014; HarvestPlus, 2015b). Biofortified crops are typically generated via selection of micronutrient accumulating traits, and there are a few means through which this can be achieved. Amongst these, one that has existed since the advent of agriculture is conventional breeding. Traditionally a long-term process requiring much investment of time and effort, advances in technology and molecular biology has since shortened the process and increased its precision when targeting specific traits. Several quantitative trait loci (QTLs) for iron accumulation have been identified in rice (Norton et al., 2010; Anuradha et al., 2012), wheat (Xu et al., 2012), maize (Jin et al., 2013), pearl millet (Kumar et al., 2016), cowpea (Santos and Boiteux, 2015), and bean (Blair et al., 2009, 2010, 2016). Already, several crops have been developed through conventional breeding under the HarvestPlus program, the most notable of which are iron biofortified pearl millet, rice and beans. The success of these biofortified crops has been demonstrated in several feeding trials. Consumption of biofortified pearl millet improved iron adsorption and iron stores in women and children (Cercamondi et al., 2013; Kodkany et al., 2013; Finkelstein et al., 2015), while biofortified rice have been found to help maintain the iron stores of non-anemic women (Haas et al., 2005). Increased iron absorption was also observed in biofortified bean meals (Petry et al., 2014, 2016). Collectively, meta-analysis of these trials indicated such biofortified crops to be particularly beneficial to iron deficient individuals (Finkelstein et al., 2017).

Despite its effectiveness, the extent to which biofortification can be done through conventional breeding is limited to the diversity in the gene pool and fertility of the species. In cases where such limitations prevail, genetic modification (GM) provides an alternative pathway. In this method, the genetic material of the host is altered in a manner that does not occur naturally. This may take the form of overexpression of a native gene, such as the OsNAS gene family in rice (Johnson et al., 2011), or expression of a foreign gene from an external source, such as the algal *FEA1* gene in cassava (Ihemere et al., 2012). A major advantage of GM is its specificity—select genes, and thus related traits, can be introduced without linkage drag that is associated with unfavorable agronomic traits. Depending on the gene combinations used, increases in iron content of up to 7.5-fold have been reported using GM technology (Trijatmiko et al., 2016).

Even with this advantage however, the release of GM food crops is controversial due to public and political concerns for environmental and human safety. Many of such concerns are directed toward herbicide-resistant GM crops. Conversely, the current direction of GM focuses more on functional foods and is more subtle, leaning toward a cis-genic rather than transgenic approach. Recent years have also seen the rapid rise of genome editing techniques—these are more specific than GM, being capable of targeting specific genome locations for modification whilst also being integration-free. In genome editing, artificial nucleases are used for targeted gene integration or deletion. Four systems have developed—meganucleases, zinc finger nucleases (ZFN), transcription factor nucleases (TALENs), and clustered regularly interspaced short palindromic repeats (CRISPR-CAS) (Sander and Joung, 2014; Bortesi and Fischer, 2015). These have been tested in a wide range of species including crops like rice and wheat, though it has yet to be applied for biofortification purposes.

To date, starchy staples that contain little micronutrients like cereals, root crops, and banana have the primary targets for iron biofortification (Namanya, 2011; HarvestPlus, 2015a; Banana21, 2016). The advantages of such targets is that they form the bulk of local diets and given proper processing, have a long shelf-life, allowing for efficient delivery of the biofortified micronutrient over a large spatial and temporal scale. A wealth of information has been generated concerning these crops as a consequence of extensive focus. Biofortification works using GM in particular, have largely concentrated on major graminaceous crops like rice, wheat, and maize. In contrast, existing studies in non-graminaceous plants were conducted mainly in model species like tobacco or *Arabidopsis* for characterization purposes. While not as extensive, some work has also been conducted in crop species like banana (Matovu, 2016), cassava (Narayanan et al., 2015) lettuce (Goto et al., 2000), and soybean (Vasconcelos et al., 2006). Given the physiological differences between the non-graminaceous and graminaceous plants, it is difficult to extrapolate the effectiveness of iron biofortification approaches in the latter to the former. As it stands, there remains much to be explored in terms of iron biofortification of non-graminaceous crops.

## Iron metabolism in plants

Prior to attempting any biofortification strategies, the significance of iron in the plants and the underlying mechanisms governing its metabolism must first be understood. Iron is the fourth most common element in the Earth's crust and can exist in a wide range of oxidation states, of which the most common are the ferrous (Fe^2+^) and ferric (Fe^3+^) forms. By virtue of its high redox potential, it forms a key component of biological processes involving electron exchange such as DNA synthesis, oxygen transport, cellular respiration, and photosynthesis, where it participates in the form of a cofactor in iron complexes. Examples of such complexes include hemoglobin, DNA helicases, and catalase.

For all its biological significance however, iron metabolic pathways can be summarized with the imagery of a precarious transfer of a radioactive material between containment facilities. Biologically, free iron may result from iron overload and/or insufficient sequestration capacity of the organism (Pietrangelo, 2003). Left alone, free Fe^2+^ catalyzes the formation of hydroxyl (·OH) radicals through the Fenton reaction, and the process repeats when Fe^2+^ is regenerated from the resultant Fe^3+^ through reduction by the superoxide radical (${\text{O}}_{2}^{-}$) (Haber and Weiss, 1934). The summation of this self-perpetuating reaction is known as the Haber-Weiss reaction:

$$\begin{array}{l} \;O_{2}^{-}\;+\;Fe^{3+}\;\to O_{2}\;+\;Fe^{2+}\\ Fe^{2+}\;+\;H_{2}O_{2}\;\to Fe^{3+}\;+\;OH^{-}+\;\cdot OH\\ O_{2}^{-}\;+\;H_{2}O_{2}\;\to \;O_{2}\;+\;OH^{-}+\;\cdot OH \end{array}$$

Reactive oxygen species (ROS) generated as a consequence of this reaction can react with cellular components to cause oxidative damage (Kehrer, 2000; Aisen et al., 2001; Papanikolaou and Pantopoulos, 2005; Jeong and Guerinot, 2009; Kobayashi and Nishizawa, 2012); however on the other hand, they also serve as important signaling molecules and are an integral part of the stress response (Apel and Hirt, 2004). The fine line between cytotoxicity and biological function, and the intimate association between iron and ROS production, highlights the significance of proper regulation of iron metabolic pathways.

Given that iron nutrition and metabolism in plants has been extensively reviewed over the years (see Table 5), this review will provide only a brief overview of the topic. Iron metabolic pathways can be divided into three main processes: uptake, translocation and storage. Despite its abundance iron has poor solubility under aerobic conditions, particularly in high pH and calcareous soils, necessitating its solubilization before uptake can occur. This process is mostly accomplished via root exudates, the composition of which varies in response to the plant's physiological state and needs. In response to iron deficiency, the plant triggers the production of factors that directly or indirectly aid iron solubilization. Enhanced concentration of glutamate, ribitol, and glucose were observed in the root exudates of iron-deficient maize, which were suggested to attract and support siderophore-producing bacterial communities to aid iron solubilization (Carvalhais et al., 2011). Notable increases were also observed in the production of organic acids like malate and citrate, which increase the availability of iron through dissolution of insoluble iron compounds (Jones et al., 1996; Sánchez-Rodríguez et al., 2014).

In addition to the aforementioned means, different plant species have adopted specific approaches toward solubilize and acquire iron. These were first categorized as Strategy I and Strategy II by Römheld and Marschner (1986) and later studies have served to cement this grouping. As the topic of iron metabolism has been extensively reviewed over the years (see Thomine and Lanquar, 2011; Kobayashi and Nishizawa, 2012; Brumbarova et al., 2015), the following sub-sections will only provide a brief overview of the process.

### Strategy I uptake mechanism

Strategy I is a reduction-based strategy used predominantly by non-graminaceous species, which includes all plants except grasses. Under iron deficiency, proton extrusion occurs through the action of H^+^-ATPases (HA), resulting in the acidification of the rhizosphere and reduction of insoluble iron (Rabotti and Zocchi, 1994; Dell'Orto et al., 2000; Santi et al., 2005; Santi and Schmidt, 2009). Phenolic compounds may also be secreted to facilitate iron uptake (Rodríguez-Celma et al., 2013). Iron and iron chelates are then reduced at the root surface by ferric-chelate reductase oxidase (FRO), which reduces Fe^3+^ chelates to Fe^2+^ by transferring electrons across the plasma membrane (Robinson et al., 1999; Waters et al., 2002). FRO is a family of membrane-bound metalloreductases that transfer electrons from cytosolic NADPH across membranes to electron-accepting substrates on the other side. In addition to facilitating acquisition of iron from the soil, this capability is also utilized in localizations where iron reduction is required for transport and/or assimilation, such as in the mesophyll (Brüggemann et al., 1993), reproductive tissues (Waters et al., 2002; Li et al., 2004a), and chloroplast membranes (Jeong and Connolly, 2009).

Following reduction at the root surface, the resulting Fe(II) ions are absorbed across the plasma membrane via the iron-regulated transporter (IRT) (Eide et al., 1996; Vert et al., 2002). IRT is a member of the *z*inc-regulated transporter, *i*ron-regulated transporter-like protein (ZIP) family that functions as membrane-bound uptake transporter for Zn and Fe (Lin et al., 2009). In Arabidopsis and tomato, IRT1 has been identified as responsible for uptake from the soil (Bereczky et al., 2003; Vert et al., 2009), with loss of function producing a severely stunted and chlorotic phenotype (Varotto et al., 2002; Vert et al., 2002). Co-regulated with IRT1 is the AtIRT2 homolog, which facilitates subcellular transport of iron and localizes to vesicle membranes instead of plasma membranes (Vert et al., 2009).

Both FRO and IRT activity is regulated in response to iron concentrations and increases in response to iron deficiency (Robinson et al., 1999; Connolly et al., 2003; Vert et al., 2003). Enhanced ferric reduction in particular, has been considered a hallmark indicator of iron deficiency (Römheld and Marschner, 1981; Higuchi et al., 1995) and increased capacity for FRO activity confers increased tolerance to low iron (Connolly et al., 2003; Peng et al., 2015).

### Strategy II uptake mechanism

Unlike Strategy I, Strategy II is used by graminaceous plants (grasses) and revolves around the use of the mugineic acid (MA) family phytosiderophores in iron acquisition. The MA biosynthetic pathway starts with the conversion of three units of S-adenosylmethionine (SAM) into nicotianamine (NA) by nicotianamine synthase (NAS) (Higuchi et al., 1994, 1999). NA is converted to a 3′ keto-intermediate by nicotianamine aminotransferase (NAAT), before being reduced to deoxymugeneic acid (DMA) by deoxymugeneic acid synthase (DMAS) (Kanazawa et al., 1994; Bashir et al., 2006). DMA can subsequently be converted to other MAs through a series of hydroxylations (Mori and Nishizawa, 1989; Ma and Nomoto, 1993), increasing levels of which improves the affinity for Fe^3+^ and chelate stability under acidic conditions (von Wirén et al., 2000). Synthesized MAs are secreted into the soil through the phytosiderophore efflux transporter TOM1 (Nozoye et al., 2011) and the resulting ferric complexes are then taken up by the roots through specialized transporters like YELLOW STRIPE 1 (YS1) and YELLOW STRIPE 1-like (YSL) (Curie et al., 2001; Murata et al., 2006; Inoue et al., 2009; Kobayashi and Nishizawa, 2012). The resulting Fe(III)-phytosiderophore complex is subsequently taken up via specialized transporters and transported throughout the plant (Kawai et al., 2001). Unlike the reduction-based approach used in Strategy I, phytosiderophore uptake is not limited by high pH, thereby conferring an advantage where such conditions are present (Römheld and Marschner, 1986).

### Translocation and storage

Following acquisition into the root symplast, iron is transported across the root to the vascular tissue and subsequently to the rest of the plant. The translocation process itself is a multi-step process, involving symplastic movement across the Casparian strip and to the desired site; the loading, unloading and transport through the vascular tissue; as well as remobilization from source tissue (Kim and Guerinot, 2007).

During transport, iron is maintained as a chelated complex with ascorbate, citrate or NA (Brown and Chaney, 1971; Stephan and Scholz, 1993; Pich et al., 1994; Grillet et al., 2014). With graminaceous plants, iron may also be complexed to DMA or MAs for transport (Koike et al., 2004; Ishimaru et al., 2010). Such complexes are pH-dependent, as are their interactions with other iron chelators (von Wirén et al., 1999). NA for instance, chelates both Fe(II) and Fe(III) at a higher pH but will preferentially bind for the former at pH 7.0. When bound to Fe(III) at equilibrium, the NA complex dominates at pH 7.0–9.0 while the structurally similar DMA complex dominates at pH 3.0–6.0. Citrate removes iron from NA at pH 5.5; and it must be converted to Fe(III)-citrate even if Fe(II) is the major form in which Fe is loaded into xylem.

As with the uptake process, non-graminaceous plants seem to rely on reduction-based strategy while graminaceous plants utilize a chelation-based one in which Fe(III) undergoes little or no change in redox state. The use of both strategies may be present in a single species, of which the only known example is rice (Ishimaru et al., 2006). This combination may represent an adaptation to the submerged conditions in which rice and its wild relatives grow, where iron is more readily available in ferrous than ferric form. Whether a similar occurrence may be found in other species remains to be seen, though orthologs of genes associated with Strategy I have also been found in other graminaceous species. An example of this is ZmIRT1 from maize, which was purported to be involved in both uptake and translocation, particularly to the seeds (Li et al., 2015). It should be noted that while differences between both strategies primarily affect the uptake process, the involvement of molecular components in the translocation process has further implications for the overall physiology of the plant.

Upon reaching the sink tissue, iron is reallocated as a cofactor in various complexes, or bound to the iron storage molecule ferritin and stored in the apoplastic space and vacuoles (Briat and Lobréaux, 1997). Subsequent translocation and remobilization may occur in response to developmental and physiological needs, such as during iron deficiency (Waters and Troupe, 2012), seed filling (Hocking and Pate, 1977; Burton et al., 1998; Garnett and Graham, 2005), senescence (Shi et al., 2012; Maillard et al., 2015), and nodulation (Strozycki et al., 2007).

## Pulses as a vehicle for biofortification

Aside from the starchy staples, another group of crops has been targeted for biofortification, albeit to a lesser degree. Pulses, as defined by the FAO (1994), are leguminous crops harvested solely for dry grain. While this term encompasses most grain legumes, soybean and peanut are excluded from this classification, because they are traditionally viewed as oilseed crops (Pulse Australia, 2016b).

Like cereals, pulses have a long history of cultivation and have been a significant constituent in human diets since around 10,000 BC (Fuller et al., 2001; Caracuta et al., 2015). As a crop, pulses present two main benefits, both of which are complementary to cereals. The first is their agronomic characteristics. By virtue of their nitrogen fixing properties, pulses are often grown as an intercrop or as a mixed crop to replenish soil nitrogen levels, thereby reducing the need for fertilizers. Cultivation with pulse crops have also been shown to increase the uptake of nitrogen, sulfur, and phosphorus by cereals, resulting in an enhanced yield and grain quality (Li et al., 2003, 2004b; Agegnehu et al., 2006; Banik et al., 2006; Gooding et al., 2007). Yield stability is also increased (Rao and Willey, 1980).

The second benefit of pulses is their nutritional density. Pulses are a rich source of carbohydrates and fiber. Their most prominent feature however, is their high protein content of 21–26% and an amino acid profile complementary to that of cereals, being rich in lysine, leucine, and arginine (Phillips, 1993; Iqbal et al., 2006; Pulse Canada, 2016). Their excellence as a vegetarian source of protein and affordability in contrast to livestock products has earned them the famous moniker of “poor man's meat.” Pulses are also rich in micronutrients like folate, thiamine, riboflavin, niacin, calcium, magnesium, iron, and zinc (Phillips, 1993; Iqbal et al., 2006; Jukanti et al., 2012). Other than contributing to macro- and micronutritional needs, several health benefits have been associated with inclusion of pulses in the diet. Their low glycemic index (GI) has been linked to the management of diabetes and diabetes-related diseases (Rizkalla et al., 2002; Sievenpiper et al., 2009) while bioactive components have been investigated for their health potential—e.g., lectins for their immunomodulatory effect, protease inhibitors for anti-inflammatory effects, and angiotensin I-converting enzyme (ACE) inhibitory peptides for their anti-hypertensive properties (Rochfort and Panozzo, 2007; Roy et al., 2010).

Despite their agronomic and nutritional benefits, pulses have not received the same amount of attention or development as the main starchy staples. Between 1961 and 2014, pulse yield and production values increased by 42.3 and 90.4%, respectively, a small fraction compared to the increase of 187.2 and 219.4% in cereals (FAO, 2016b). Much of this disparity can be attributed to developments made during the Green Revolution, in which the focus on productivity and protein-calorie malnutrition led to the shift from cultivation of traditional micronutrient-rich crops to the more productive and profitable starchy cereals (Pinstrup-Andersen and Hazell, 1985; Pingali, 2012). Poor policy and diversion of land to cereal cultivation has led to a reduction in pulse supply, effectively driving prices up and decreasing consumption per capita (Kennedy and Bouis, 1993; Kataki, 2002; Akibode and Maredia, 2012).

**Figure 1:**
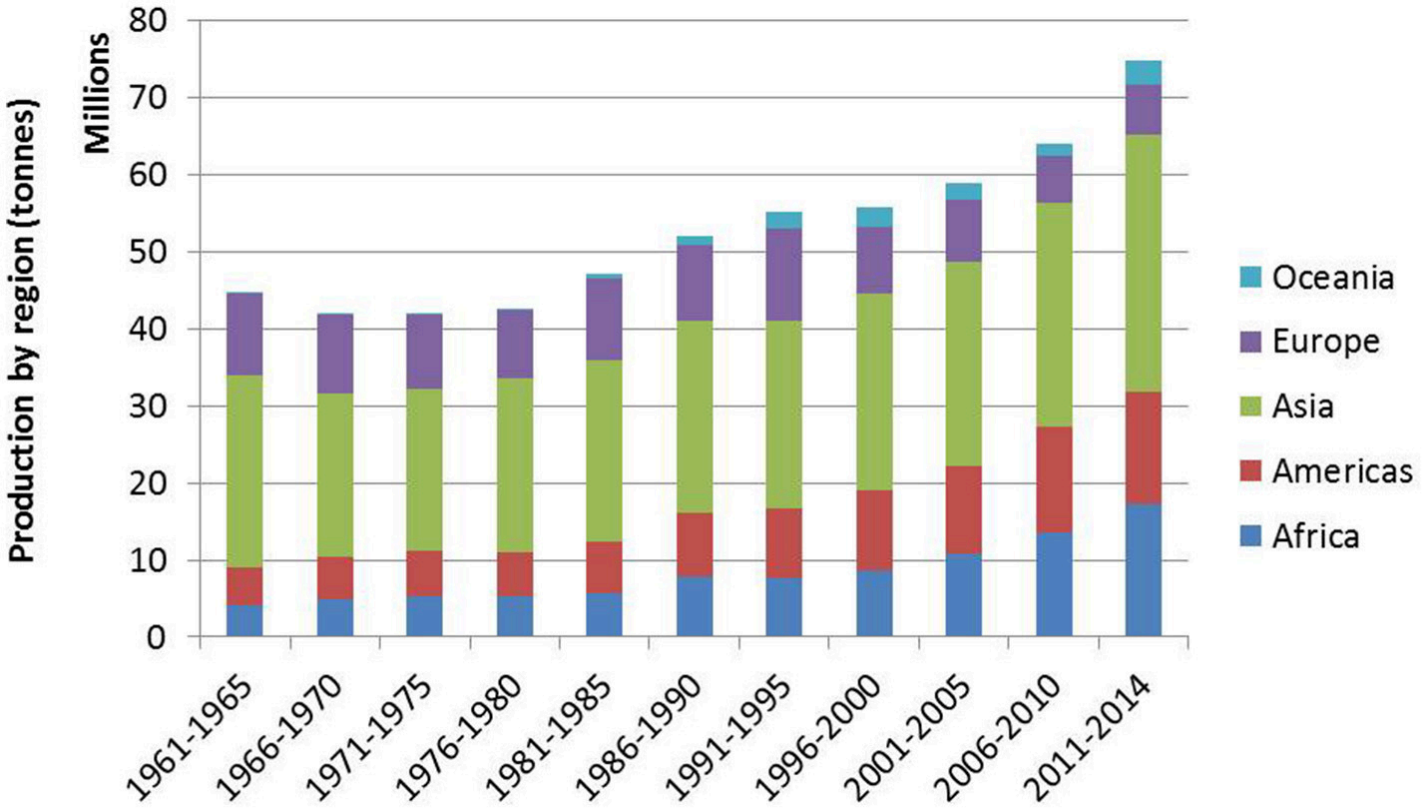
Average pulse production by region (FAO, 2016a).

As highlighted in the special feature on pulses in the 2014 Food Outlook (FAO, 2014), recent years have seen several key changes in pulse production and trade. Asia remains the region with the highest pulse production, with India continuing as the largest pulse producing country, contributing at least 20% toward global pulse production (Figure 1 and Table 4). Production in other regions except Europe has also increased, fueled by domestic and international demand. In contrast to these countries is China, whose production has decreased due to a number of factors such as population increase and decreasing availability of arable land. Despite the shift in preference for animal-based products and protein that accompanies growing affluence, India and China remain major importers, consuming approximately 40% of the world's pulse production as food, and 30% as feed. Much of this is provided by major exporters like Canada and Australia. With other major producers like Myanmar and Brazil, pulse consumption is primarily domestic.

**Table 4:** Growth in production of major pulse producers (FAO, 2014, 2016b).

	**Production (mmt)**				
**Country**	**1961**	**1981**	**2001**	**2014**	**Principal pulse crops**
India	12.9	10.8	12.2	19.98	Chickpeas, beans, pigeon peas
Myanmar	0.2	0.4	2.0	5.0	Beans, pigeon peas, chickpeas
Canada	0.1	0.2	3.4	5.8	Peas, lentils
China	8.5	6.4	5.1	4.5	Beans, broad beans, peas
Brazil	1.8	2.4	2.5	3.3	Beans
Nigeria	5	0.6	2.3	2.2	Cowpeas
Ethiopia	0.6	0.9	1.2	2.6	Broad beans, beans, chickpeas, peas
Australia	0	0.3	2.7	3.0	Lupines, lentils, chickpeas
USA	1.1	1.7	1.3	2.4	Beans, peas
Tanzania, U. Rep.	0.1	0.3	0.8	1.8	Beans
Rest of the world	15.0	17.5	22.6	27.02	
Total	40.8	41.6	55.9	77.6	

**Table 5:** List of general reviews on iron nutrition and metabolism.

• Hell and Stephan, 2003
• Kim and Guerinot, 2007
• Walker and Connolly, 2008
• Morrissey and Guerinot, 2009
• Thomine and Lanquar, 2011
• Kobayashi and Nishizawa, 2012
• Thomine and Vert, 2013
• Briat et al., 2015
• Brumbarova et al., 2015
• Connorton et al., 2017
• Curie and Mari, 2017

Pulse production, consumption, and trade are expected to increase alongside population growth, particularly with increasing promotion from government campaigns (Akibode and Maredia, 2012) and the declaration of 2016 as the “International Year of Pulses” by the UN General Assembly. Increasing awareness and concern over nutritional composition of food, particularly by food manufacturers, has attracted greater interest in pulses, which will likely translate into further support and development of the pulse industry (FAO, 2014).

## Challenges of pulse biofortification

Concerning the biofortification of pulses however, there are some challenges. While pulses are a diverse group featuring a wide variety of species and cultivars, this has presented itself as a double-edged sword. The genetic richness is a treasure trove that lends itself to crop improvement, but has simultaneously resulted in a lack of a concerted global effort at development. Development has also thus far has been focused on yield, disease tolerance, and macronutrient quality, with little to no emphasis on other nutritional aspects. However, this has changed over the past decade with the growing interest in micronutrient content. Increasing numbers of genotypes and cultivars being assayed for their iron and zinc composition (Ray et al., 2014; Thavarajah et al., 2014; Santos and Boiteux, 2015; Blair et al., 2016). Concerning outputs however, there has been little published work on pulse biofortification, with even fewer examples of biofortified pulses. Currently the only known example of a biofortified pulse is the high iron common bean generated from the HarvestPlus breeding program; to date, several varieties have been produced with improvements in iron content ranging from 47 to 94% (Katsvairo, 2015).

Another challenge with pulse biofortification is the bioavailability of iron. Bioavailability, as defined by Carpenter and Mahoney (1992), is the “proportion of a nutrient present in food that the body is able to absorb and utilize by incorporation into physiologically functional pools.” It is a complex and multifaceted issue subject to a range of physiological and physiochemical factors such as those illustrated in Table 6. In humans, iron uptake occurs in the duodenum and upper jejunum in the intestinal tract. Regulation is done via absorption rather than excretion, and occurs in response to iron status and rate of erythropoiesis (Wheby et al., 1964; Bothwell, 1995). Under normal conditions, absorption is inversely correlated to the state of the body's iron stores with increased uptake by the intestinal mucosal cells as iron stores are depleted (Walters et al., 1975).

**Table 6:** Factors affecting bioavailability of some trace elements (House, 1999).

**Host factors**	**Dietary factors**	
	**Dietary composition**	**Food preparation**
Age Sex Ethnic background ◦ Types of food selected ◦ Geographic living area Economic status ◦ Type, quality, and quantity of selected food Physiological status ◦ Pregnancy ◦ Lactation ◦ Physical activity Nutritional status ◦ Moderate or frank deficiency ◦ Lean body mass Disease (including parasitism)	Protein quality ◦ Protein source ◦ Animal vs. plant protein ◦ Amino acid balance Protein quantity Trace element quantity Physiochemical form of trace element Nutrient interactions ◦ Element–element ◦ Element–organic compounds Promoters ◦ Meat ◦ Ascorbate ◦ Citrate ◦ Vitamin D ◦ Some amino acids ◦ Some sugars Inhibitors ◦ Phytate ◦ Oxalate ◦ Polyphenols ◦ Fiber ◦ Goitrogens ◦ Excess ascorbate or folate Micronutrient deficiencies ◦ Ascorbate ◦ Riboflavin ◦ Vitamin E	Raw Cooking (various methods) Fermentation Malting Milling Extraction Soaking

For uptake to occur however, the mineral must be in a form absorbable by the mucosal cells. Dietary iron can be classified as heme or non-heme based on its attachment to heme proteins or lack thereof. Both form separate pools in the gastrointestinal tract and undergo different absorption pathways (Björn-Rasmussen et al., 1974). Compared to its heme counterpart, non-heme iron is the predominant form found in plants and has a lower bioavailability and is particularly susceptible to the influence of dietary factors (Björn-Rasmussen et al., 1974). This is of especial significance in pulses due to the abundance of naturally occurring inhibitors which bind to iron and prevent uptake. Examples of such inhibitors include phytic acid, polyphenols, tannins, and fiber (Sandberg, 2002; Ghavidel and Prakash, 2007; Thavarajah et al., 2014).

Phytic acid (PA), also known as inositol hexaphosphate or IP6, has been identified as a one of the major inhibitors of iron bioavailability. PA serves as the principal form of phosphorus storage in seeds, where it is present as a phytate salt of mineral cations like potassium, magnesium, calcium, manganese, and zinc. Depending on the species, cultivar and conditions of growth, it may constitute 40–84% of total seed phosphorus (Lolas et al., 1976; Griffiths and Thomas, 1981; Ravindran et al., 1994). Amongst the inositol polyphosphates, the lower inositol phosphates IP2, IP3, and IP4 play a minor role in inhibiting iron bioavailability (Sandberg et al., 1989, 1999). The main inhibitors are IP6 and IP5, which are capable of reducing iron solubility by 38.8 and 33%, respectively, through the formation of insoluble complexes (Sandberg et al., 1989).

The impact of PA on biofortification efforts can be illustrated using the example of the biofortified beans. Despite their higher iron content, feeding trials conducted in Rwanda have indicated iron bioavailability of biofortified beans was similar, if not lower, than that of the unfortified beans (Petry et al., 2012, 2014). As a result, while the total of iron absorbed from the biofortified beans was higher than the unfortified beans, it was considerably less than expected. As a means to improve the effectiveness of biofortification, the reduction in PA concentration was recommended by the authors (Petry et al., 2012, 2014).

This recommendation has been applied in several cereal crops (Larson et al., 1998, 2000; Raboy et al., 2000; Guttieri et al., 2004) and more recently in bean (Campion et al., 2009). The effectiveness of the low-phytic acid bean lines is currently inconclusive however, as bioavailability assessments have yielded conflicting results due to differences in experimental design (Petry et al., 2013, 2016). Poor cooking quality was also observed in the low-phytic acid seeds, which may have contributed the adverse gastrointestinal side-effects in the participants in one of the studies (Petry et al., 2016). The relationship between phytic acid and cooking quality have been alluded to in other studies on lentil and bean (Kon and Sanchuck, 1981; Bhatty and Slinkard, 1989). Interestingly, no such effect was reported in low-phytic acid maize lines (Mendoza et al., 1998); whether this is a legume-specific issue remains to be confirmed. Aside from influencing cooking quality, phytic acid is also known to have antioxidant properties and protective effects against heart disease and cancer (Sharma, 1986; Nelson et al., 1988; Vucenik and Shamsuddin, 2003). It is unknown if reduction in phytic acid content would affect such properties. Similarly, the subsequent long-term effect on human health is unknown.

For all the challenges presented in this section however, there is much potential to be explored in pulse biofortification. Much of the existing knowledge concerning this area is limited to the work done on the iron biofortified bean. That has proven to be a successful means of alleviating iron deficiency, promising much for other pulses.

## Chickpea as a target for biofortification

Chickpea (*Cicer arietinum*) is an important pulse crop that has been cultivated by humans since the Stone Age. As of 2009, it is the second most important pulse crop in the world after the common bean, having overtaken peas as the pulse crop with the second highest global production values. Global production has climbed steadily since 2008 to exceed 14.2 million tons in 2014, of which approximately 96% is grown in developing countries (FAO, 2016b). India in particular, has historically been the largest producer and consumer of chickpea; in 2013 alone it contributed approximately 65 and 33% to total chickpea production and import, respectively (FAO, 2016b). In terms of consumption, it is difficult to obtain precise statistics due to the lack of available data. However, based on calculations using production and trade values, the global average for chickpea consumption was estimated to be around 1.3 kg/year per person between 2006 and 2008, with South Asia and the Middle East-North Africa regions being the biggest consumers at 4.25 kg/person and 2.11 kg/person per year, respectively (Akibode and Maredia, 2012). The demand is predicted to grow, particularly in Africa and Asia, due to population increase and increasing support from the governments in encouraging pulse consumption (Rao et al., 2010; Akibode and Maredia, 2012). This increase in demand is not limited to those regions; in the USA for instance, net domestic use of chickpea nearly doubled from 199.6 g in 2010 to 322.1 g in 2014 (Wells, 2016).

Most of the chickpea in the global market can be classified into two main types which are primarily distinguishable by their seed morphology, specific aspects of which influence their end-use. The first type is the kabuli, also known as garbanzos. Kabuli seeds are large and round, weighing approximately 400 mg per seed (Pulse Australia, 2016a). The seed coat is thin and light-colored, ranging from shades of white to cream and the seeds are typically consumed whole or made into hummus (Gaur et al., 2015; Pulse Australia, 2016a). Kabuli cultivation areas are mostly located in Southern Europe, Northern Africa, Afghanistan, Pakistan, Chile, and India (Gaur et al., 2015).

The second type is the desi, which forms the bulk of the international export market (Rao et al., 2010). Desi seeds are small, wrinkled, and angular, with an approximate weight of 120 mg per seed (Pulse Australia, 2016a). The seed coat is also 1.2 to 3 times thicker than the kabuli (Umaid et al., 1984; Wood et al., 2011) and can be found in a greater variety of colors ranging from brown to yellow, as well as orange, black and green. Desi seeds are commonly dehulled and split to obtain the cotyledons, which are then known as chana dhal and can in turn be milled to flour, known as besan or gram flour.

As a food crop, chickpea can be utilized in a variety of ways. Green pods, immature seeds and young leaves can be consumed as a vegetable while the stover and pod husks can be used as animal feed (Ibrikci et al., 2003; Yadav et al., 2007). The primary commodity however, is the dried mature seed which can be used as animal feed or for human consumption. With the latter, the long history of consumption in various regions such as India, the Middle East, and Europe has given rise to a diversity of dishes in which chickpea can be utilized. Chickpeas are consumed on their own or with other foods; seeds may be eaten whole, hulled, or ground into flour from which other products may be derived. Preparation for consumption can be by various processing methods such as soaking, sprouting, fermenting, boiling, steaming, roasting, extrusion, and puffing (Yadav et al., 2007), all of which exert different effects on the overall nutritional quality (Poltronieri et al., 2000; Sebastiá et al., 2001; Ghavidel and Prakash, 2007; Hemalatha et al., 2007a).

Much like other pulses, the nutritional qualities of chickpea have long been recognized and documented. In addition to high protein content (20–22%), chickpeas are also rich in micronutrients like folate, magnesium, zinc, and iron (USDA, 2013). Studies conducted by different authors have found iron content to range from 2.4 to 11 mg/100 g (e.g., USDA^1^; Meiners et al., 1976; Wood and Grusak, 2007). Likewise, various studies have reported differing values for phytic acid and other antinutrients (e.g., Chitra et al., 1995; Ghavidel and Prakash, 2007; Hemalatha et al., 2007b), indicating a possible effect of genotype and environmental factors on overall iron bioavailability. When measured as dialyzable iron generated from a simulated gastrointestinal digest, bioavailability has been found to vary widely across different studies, ranging from about 6 to 25% (Chitra et al., 1997; Ghavidel and Prakash, 2007; Hemalatha et al., 2007b). The reason behind this disparity is as yet unclear, though analytical procedures and variations in samples have been suggested as a possible cause (Platel and Srinivasan, 2016). Given the multifaceted nature of nutrient bioavailability, the values obtained are at best relative.

Regardless of processing methods and culinary adjustments, the composition of the starting material is vital. As demonstrated by Petry et al. (2012), enhancement of iron content alone does not necessarily translate into an improved iron status of the consumer, particularly when there is a concurrent enhancement in phytate content. Both the iron content and overall composition of the grain itself should therefore be considered in biofortification strategies. However, in light of the lack of information concerning bioavailability, it would be prudent for biofortification efforts to first target total seed iron content before progressing to bioavailability. Considerable progress has been made to that end, particularly with the growing interest in chickpea as a target for iron biofortification. While a concerted global effort has yet to materialize, pockets of development have emerged with India and Canada at the forefront. To date, the chickpea genome has been sequenced (Varshney et al., 2013). Chickpea populations in those countries have also been screened for genetic diversity and iron accumulation traits, allowing for identification of the associated QTLs (Diapari et al., 2014; Upadhyaya et al., 2016). In terms of biofortification via GM, no work has been done yet. It is however, a viable option—while chickpea can be considered a recalcitrant species, successful transformation protocols have been established (Sarmah et al., 2004; Indurker et al., 2010). Such work would also provide insight into the physiological workings; this in turn can inform later biofortification efforts by identifying specific traits or mechanisms which can be targeted by breeding or GM.

Given the relative youth of this endeavor to biofortify chickpea for iron, no biofortification targets have yet been set. As stated by Bouis and Welch (2010), several variables need be considered in the setting of such targets. The challenge lies primarily in the lack of information concerning the different variables in chickpea. Unlike the common bean which serves as a staple, chickpea is a secondary staple and depending on the type and cultivar, may be processed into various forms for consumption (Yadav et al., 2007). This would in turn affect iron content and bioavailability. Consequently, the consumption profile for chickpea is expected to be lower and potentially more varied compared to the common bean, particularly across different age and cultural demographics. Until more detailed and specific information concerning chickpea is obtained, only general assumptions may be made. In the interim however, efforts can be made to understand and engineer for increased iron content.

## Potential approaches to engineering for enhanced iron content in chickpea

In the interest of iron biofortification, five rate-limiting steps to grain iron accumulation have been identified by Sperotto et al. (2012): (1) uptake from soil, (2) xylem loading in roots, (3) phloem transport from leaves, (4) unloading for grain filling, and (5) grain sink strength. These steps can be classified into the three main processes of uptake, translocation, and storage. Genes associated with these processes have been identified as promising candidates for iron biofortification, and over the past decade several of them have been applied to different plant species.

### Rice—a case study

Amongst these, rice can be considered the flagship for transgenic iron biofortification. Since rice is a grain crop, the lessons learnt may, in part, be transferrable to chickpea. As illustrated in the review by Masuda et al. (2013a), several gene combinations targeting the uptake, translocation, storage or any combination of the three processes have been attempted to differing levels of success. Of the combinations investigated thus far, those containing NAS and ferritin (FER) have yielded the most promising results. Individually, NAS and FER have been demonstrated to enhance iron accumulation. Overexpression of the former for instance, increased iron content in polished grains by four-fold (Johnson et al., 2011), while overexpression of a soy homolog of the latter produced up to a 3.7-fold increase (Vasconcelos et al., 2003). Similar results have been obtained when combined with other genes involved in iron transport or MAs synthesis. Approximately three-fold increase was obtained from overexpression of the rice yellow stripe like-2 (YSL2), barley NAS1, and soybean FER genes (Masuda et al., 2012). A four-fold increase was obtained from overexpressing soybean FER in conjunction with barley NAS1, two NAAT genes and a mugineic acid synthase gene (Masuda et al., 2013b).

The best results however, were achieved simply by using just the NAS-FER combination. Constitutive expression of *Arabidopsis* NAS1 together with barley FER and a fungal phytase, both under the regulation of a rice seed storage globulin promoter, enhanced iron accumulation in rice endosperm by up to six times (Wirth et al., 2009). More recently, up to 7.5-fold increase in iron content was obtained from transgenic rice through constitutive overexpression of OsNAS2 and seed-specific expression of soybean FER (Trijatmiko et al., 2016). The enhancement was not limited to total iron content alone, as iron bioavailability was similarly improved. This was demonstrated in studies with Caco-2 cell cultures, where the amount of iron absorbed from the transgenic lines was more than double that of the controls, even in the absence of any bioavailability enhancers (Trijatmiko et al., 2016).

### Tailoring a GM approach to chickpea

Whether a similar feat may be emulated in other species is yet unknown as the NAS-FER gene combination has only been applied in rice. In this case, the success of the NAS-FER gene combination may be attributed to the unique role of NA in the non-graminaceous system. In it, NA, by virtue of its in role in the synthesis of DMA and MAs, is directly involved in the uptake and translocation processes (Wang et al., 2013). Through combination with FER, it allows for the simultaneous targeting of all three major processes of iron metabolism, thereby overcoming the rate-limiting steps listed by Sperotto et al. (2012). Incidentally, the issue of bioavailability is also resolved—NA is a known enhancer of iron bioavailability (Zheng et al., 2010), while ferritin has a bioavailability equivalent to that of ferrous sulfate which is used in iron supplements (Davila-Hicks et al., 2004; Lönnerdal et al., 2006).

Based on these observations, there is reason to believe that application of the NAS-FER approach to other graminaceous species will yield similar outcomes to rice. The effect however, may be limited in non-graminaceous crops like chickpea due to the absence of the MAs biosynthetic pathway, which diminishes the contribution of NAS to the uptake process. Such is evident when comparing the results of NAS overexpression in graminaceous and non-graminaceous species, where greater enhancement in iron content was observed in the former (Masuda et al., 2009; Johnson et al., 2011) than the latter (Douchkov et al., 2005; Cassin et al., 2009). In any case, prior studies in model species like *Arabidopsis* and tobacco have confirmed the individual effect of each gene (Van Wuytswinkel et al., 1999; Douchkov et al., 2005; Cassin et al., 2009); should the NAS-FER approach be applied to chickpea, some enhancement of seed iron content can still be expected. Similarly, iron bioavailability can increase, though the extent is difficult to predict given the higher levels of inhibitors in chickpeas compared to rice (Hemalatha et al., 2007b).

At the moment these are speculations—with the existing transgenic research concentrated on cereals, there is little precedent for reliable extrapolation to a leguminous crop. Nonetheless, two main principles can be drawn from the success of the NAS-FER strategy in rice, and that is (1) the simultaneous targeting of multiple rate-limiting steps, and 2) targeting of bioavailability in addition to iron content.

#### Targeting multiple rate-limiting steps

One of the main challenges to engineering for iron accumulation in chickpea is the lack of specific knowledge on iron homeostasis in chickpea. Even amongst closely related members of non-Gramineae, interspecies variation exists as different mechanisms or components may be favored. Such is evident when comparing studies on QTLS and iron accumulation traits—differing suites of associated genes have been found in soybean (Ning et al., 2015) and chickpea (Upadhyaya et al., 2016).

Examination of such genes may yield potential candidates for use in GM biofortification. Drawing from the example of NAS in rice, selection of such candidate genes can be based on their involvement in both uptake and translocation processes, though extra emphasis should be placed on the former. As mentioned by Sperotto et al. (2012), the ability to access soil iron under differing environmental conditions is the first major bottleneck to iron accumulation in plants, with poor uptake capacity typically associated with susceptibility to iron deficiency (Mahmoudi et al., 2007; Waters and Troupe, 2012).

Several genes fitting such criteria can be found in the list by Upadhyaya et al. (2016), and key examples include transporters like IRT, FRO, YSL, NRAMP (natural resistance-associated macrophage protein), and zinc-regulated transporter, iron-regulated transporter-like protein (ZIP). As these are involved in both uptake and translocation (Vert et al., 2002; Lanquar et al., 2005; Vasconcelos et al., 2006), targeting them may, theoretically, allow for two processes to be simultaneously enhanced. Constitutive overexpression of AtFRO2 in soybean for instance, was found to increase both iron uptake and leaf iron content (Vasconcelos et al., 2006). However, this effect may be dependent on the homolog used as each may serve specific functions. Within FRO family in *Arabidopsis* for instance, AtFRO2 is expressed in the roots and facilitates uptake during iron deficiency (Connolly et al., 2003), while FRO7 is expressed in the chloroplasts where it contributes to iron supply (Jeong et al., 2008).

A potential pitfall to such an approach is the inherent regulatory mechanisms. Unlike NAS, which appears to be amenable to manipulation with little to no side-effects (Pianelli et al., 2005; Johnson et al., 2011; Lee et al., 2012), transporters like IRT and FRO appear to be regulated by mechanisms which are less forgiving to interference. In *Arabidopsis* overexpressing AtIRT1 or AtFRO2, no increase in root reduction or protein levels was observed under iron-sufficient conditions due to post-transcriptional regulation (Connolly et al., 2002, 2003). However, no such impediment was noted when the maize homolog, ZmIRT1, was overexpressed, with transgenic lines having significantly higher seed iron contents compared to the wild-type (Li et al., 2015). This discrepancy was attributed to the low homology between the ZmIRT1 and the native IRT genes, which may in turn point to a means of bypassing post-transcriptional regulation.

Aside from enhancing uptake and translocation, sink strength may also be targeted. As far as GM biofortification efforts go, this has traditionally been done using FER. The application in chickpea appears to be highly feasible as amongst the fifteen genes associated with seed iron accumulation in chickpea, two were identified as ferritins. Strong constitutive FER overexpression however, carries the risk of excessive iron sequestration, resulting in manifestation iron deficiency symptoms (Van Wuytswinkel et al., 1999). This may be avoided through seed-specific expression, particularly in the cotyledons which are the main products. Much like the NAS-FER approach in rice, a multigenic approach combining FER with one of the aforementioned transporters may also be used. This will also allow for simultaneous targeting of all three major processes of iron metabolism, thereby overcoming the bottlenecks described by Sperotto et al. (2012). Theoretically, such a multigenic approach may also translate to higher levels of iron accumulation through synergy between the transgenes. Such was observed in rice, where the NAS-FER combination produced a synergistic effect (Wirth et al., 2009; Trijatmiko et al., 2016), resulting in higher seed iron contents compared to the monogenic NAS approach (Masuda et al., 2009; Johnson et al., 2011; Lee et al., 2011).

#### Targeting bioavailability

As previously mentioned, the use of FER has the added benefit of enhancing iron bioavailability in addition to total iron content. Concerning the enhancement of bioavailability however, the use of FER is but one means. Other options may include targeting the concentrations of inhibitors or enhancers. The former has already been attempted in maize and rice through the overexpression of phytase, and increases in bioavailability have been reported (Lucca et al., 2001; Drakakaki et al., 2005; Wirth et al., 2009). While promising, the actual effect on human health is unknown as the assays were done using *in vitro* methods. Given that no negative consequences were observed from the addition of exogenous phytase to food (Hurrell et al., 2003), it is likely that the detrimental effects observed with low-phytate beans (Petry et al., 2016) may be avoided.

Concerning bioavailability enhancers, some work has already been done in the form of NAS-overexpressing crops and the results discussed in the above sections (Johnson et al., 2011; Trijatmiko et al., 2016). An alternative candidate is ascorbic acid, a potent enhancer occurring naturally in plants which has been demonstrated to prevent the inhibitory effects of phytate and polyphenols (Hallberg et al., 1989; Siegenberg et al., 1991). However, while promising, ascorbic acid is also infamous for its thermal instability (Van den Broeck et al., 1998; Munyaka et al., 2010), with cooking generally resulting in degradation (Sood and Malhotra, 2002; Moriyama and Oba, 2008). It's effectiveness in a transgenic biofortification strategy is therefore questionable given the processing requirements of a grain crop like chickpea.

## Summary and implications

In summary, iron deficiency is a global health problem which may be alleviated through the use of biofortified crops. Transgenic biofortification efforts, as well as most studies on iron metabolism, thus far have largely been directed at cereal crops like rice. As members of the Gramineae family, their molecular biology and physiology differ significantly from their non-graminaceous counterparts. Consequently, biofortification strategies successfully applied in a graminaceous species like rice may behave differently in a non-graminaceous species. The extent of this difference is currently unclear as studies have primarily been performed in model species like *Arabidopsis* and tobacco primarily for gene characterization. There is a need to tailor specific biofortification strategies for use in non-graminaceous species. This is particularly so for important secondary staples like pulses—population growth as well as environmental pressures has increased the demand for affordable, water-efficient sources of protein. As the second most important pulse crop in the world, chickpea stands in a unique position to meet this need. It is widely consumed in the Asian and African regions where population growth, as well as the incidence of iron deficiency, is highest. The iron biofortification of chickpea can therefore serve as a sustainable means to alleviate the public health burden where it is heaviest.

While some breeding work is currently underway, there has been no recorded attempts to biofortify chickpea via a GM approach. However, the avenue to do so is available, given the establishment of successful transformation protocols. Valuable lessons can be learnt from the success of the GM biofortified rice and applied to the formulation of biofortification strategies for pulse crops like chickpea. Existing QTL and trait analysis have identified several candidate genes which may be used to enhance iron content and/or bioavailability, opening up new doors for further exploration.

## Author contributions

This review was part of a larger project designed and headed by SM, BW, AJ, and SD. The document was written by GT, TMLH and MRK. Drafts were edited by the other authors, and upon their approval, was submitted for publication.

### Conflict of interest statement

The authors declare that the research was conducted in the absence of any commercial or financial relationships that could be construed as a potential conflict of interest.

## Footnotes

^1^USDA Basic Report: 16056, *Chickpeas (Garbanzo Beans, Bengal Gram), Mature Seeds, Raw*.
